# A virtual mother-infant postpartum psychotherapy group for mothers with a history of adverse childhood experiences: open-label feasibility study

**DOI:** 10.1186/s12888-023-05444-x

**Published:** 2023-12-18

**Authors:** Elisabeth Wright, Jovana Martinovic, Diane de Camps Meschino, Lucy C Barker, Diane A Philipp, Aliza Israel, Neesha Hussain-Shamsy, Geetha Mukerji, Vivienne Wang, Antara Chatterjee, Simone N Vigod

**Affiliations:** 1 Department of Psychiatry, University of Toronto, Toronto, ON M5T 1R8 Canada; 2 Institute for Health Policy, Management and Evaluation, University of Toronto, Toronto, ON M5T 3M6 Canada; 3 Department of Psychiatry, Women’s College Hospital and Research Institute, 76 Grenville Street, Toronto, ON M5S 1B2 Canada; 4 Department of Medicine, Temerty Faculty of Medicine, Sunnybrook Health Sciences Center, King’s College Circle, University of Toronto, Toronto, ON Canada; 5 Women’s College Hospital Institute for Health System Solutions and Virtual Care, 76 Grenville Street, Toronto, ON Canada; 6 Garry Hurvitz Centre for Community Mental Health at Sickkids, Toronto, ON Canada

**Keywords:** Maternal-child, Mental health, Adverse childhood experiences

## Abstract

**Objectives:**

Mothers with a history of adverse childhood experiences (ACE) are at elevated risk for postpartum mental illness and impairment in the mother-infant relationship. Interventions attending to maternal-infant interactions may improve outcomes for these parents and their children, but barriers to accessing in-person postpartum care limit uptake. We adapted a postpartum psychotherapy group for mothers with mental illness (e.g., mood, anxiety, trauma-related disorders) and ACE for live video-based delivery, and evaluated feasibility, acceptability, and preliminary efficacy in an open-label pilot study.

**Methods:**

We recruited adults with children (6–18 months) from a perinatal psychiatry program in Toronto, Canada. The intervention was a live video-based 12-week interactive psychotherapy group focused on maternal symptoms and maternal-infant relationships. The primary outcome was feasibility, including feasibility of recruitment and retention, fidelity of the intervention, and acceptability to patients and group providers. Maternal clinical outcomes were compared pre- to post-intervention, as secondary outcomes.

**Results:**

We recruited 31 participants (mean age 36.5 years (SD 3.9)) into 6 groups; 93.6% (n = 29) completed post-group questionnaires, and n = 20 completed an optional post-group acceptability interview. Mean weekly group attendance was 83% (IQR 80–87); one participant (3.2%) dropped out. All group components were implemented as planned, except for dyadic exercises where facilitator observation of dyads was replaced with unobserved mother-infant exercises followed by in-group reflection. Participant acceptability was high (100% indicated the virtual group was easy to access, beneficial, and reduced barriers to care). Mean maternal depressive [*Edinburgh Postnatal Depression Scale: 14.6 (SD 4.2) vs. 11.8 (SD 4.2), paired t, p = 0.005]* and post-traumatic stress [*Posttraumatic Stress Disorder Checklist for DSM-5*: *35.5 (SD 19.0) vs. 27.1 (SD 16.7)], paired t, p = 0.01]* symptoms were significantly lower post vs. pre-group. No differences were observed on mean measures of anxiety, emotion regulation or parenting stress.

**Conclusions:**

Recruitment and retention met a priori feasibility criteria. There were significant pre- to post-group reductions in maternal depressive and post-traumatic symptoms, supporting proceeding to larger-scale implementation and evaluation of the intervention, with adaptation of dyadic exercises.

**Supplementary Information:**

The online version contains supplementary material available at 10.1186/s12888-023-05444-x.

## Background

Postpartum mental illnesses are common, with depression alone affecting an estimated 13% of mothers following childbirth [1]. Effective treatment of postpartum mental illness is essential to optimize maternal health and child developmental outcomes [2, 3]. Maternal history of adverse childhood experiences (ACE) increases risk for perinatal mental illness including depression, anxiety, and trauma- and stressor-related disorders [4–9], for more persistent and severe postpartum symptom trajectory [5, 10–12] and for greater residual symptoms following standard mental health treatments [12]. Maternal ACE is also linked with impairments in the mother-infant relationship including reduced parenting sensitivity, bonding, and attachment security [6, 13–15], and negative developmental outcomes for infants [5, 16, 17].

Given the high prevalence of ACE among those with postpartum mental illness, interventions addressing the combined impact of postpartum mental illness and ACE on the maternal-infant relationship are crucial. In general, psychotherapeutic interventions are effective in treating postpartum mental illness [18, 19]. Yet, those that focus solely on treating maternal symptoms often do not adequately improve maternal-infant relationships or infant development [20, 21]. Postpartum psychotherapeutic interventions that attend specifically to the maternal-infant relationship demonstrate improvements in maternal-infant responsiveness, infant attachment security and socio-emotional competence [22–24].

To date, most interventions in this area have been delivered in an in-person format. However, practical challenges of scheduling with an infant, finding appropriate childcare, maternal and infant illnesses, and arranging transportation have been cited as barriers to participation in in-person therapy [25, 26]. The SARS-CoV2 pandemic created an additional barrier to safe in-person care, especially in group settings. Virtual models of health represent an opportunity to address some of the postpartum barriers noted above, as well as difficulties related to the impact of depression (e.g., low energy and motivation) and anxiety (e.g., avoidance) on attendance. While some psychotherapeutic components used in postpartum psychotherapy groups have shown effectiveness in virtual administration, no professionally-facilitated psychotherapy group addressing postpartum mental illness and the maternal-child relationship in the context of maternal ACE has been evaluated virtually. This gap in service delivery, if filled, could significantly improve treatment uptake for maternal mental illness and reach those in the highest risk groups.

Members of our team previously implemented an in-person maternal-infant postpartum psychotherapy group for mothers with ACE based on the principle of dual-focus on maternal symptomatology and maternal-infant relationship, delivered in group format to increase resource efficiency and reduce isolation [26]. We adapted this therapy group for live video-based delivery, such that group members could participate using secure video-visit technology from their homes. Herein, we evaluated the feasibility of implementation, acceptability, and preliminary efficacy of the live video-based intervention in an open-label pilot study.

## Methods

### Design and setting

This was a single site open-label feasibility trial conducted from July 2021-June 2022 within a multidisciplinary ambulatory hospital-based perinatal psychiatry program in Toronto, Canada, publicly funded through the provincial health care plan. Within the program, psychotherapy is delivered by highly-trained social worker, registered psychotherapist, and psychiatrist therapists. Referrals to the study were made from within the clinical program, either by the assessing psychiatrist, or by another program clinician upon completion of another psychotherapy modality. Potentially interested participants were contacted by phone by a trained research coordinator/assistant who explained study requirements, obtained written informed consent, and conducted eligibility assessments. Data were collected through the clinical patient chart, and directly from participants via online REDCap™ surveys and qualitative interviews. We aimed to optimize diversity across demographic variables when selecting participants for interviews. We randomly selected 3–4 participants from the first group cohorts for invitation to interview. After these initial interviews were conducted, we examined the demographics of interviewed participants and compared them with demographics of the total participant population, and subsequently invited participants for later interviews to increase diversity across demographic variables. We expected to reach saturation at around 20 participants, which was confirmed. Qualitative interviews were conducted by phone by one team member (EW) an average of 22 days following group completion (range 2–43 days post-group). Participants were invited by a research team member who was not involved in group facilitation, to attend an approximately 45-minute phone interview to provide feedback about their experience in the group. All interviewees were asked the pre-determined questions in the same order (Appendix D), including an open-ended question (#11) to provide any thoughts or feedback not covered in the fixed questions.

Approval for this study was obtained through Women’s College Hospital Ethics Assessment Process for Quality Improvement Projects (WCH APQIP). Written informed consent was obtained from all participants.

### Participants

We included individuals: (1) self-identifying as a mother (inclusive of cisgender and transgender women and non-binary individuals), with (2) an infant between 6 and 18 months of age (adoptive and birth parents); (3) a diagnosis of a mood, anxiety, or trauma/stressor-related disorder (by program psychiatrist); (4) a history of one or more adverse childhood experiences, defined as a self-reported history of childhood trauma (i.e., physical, psychological or sexual trauma, or neglect), as obtained by clinical interview; and (5) evaluated as appropriate for the group by one of the facilitating clinicians. We excluded those with: (1) active alcohol or substance use disorder in the previous 12 months; (2) active suicidal ideation, mania or psychosis; (3) incapacity to consent to treatment; (4) and inability to speak/understand English. Participants were required to be physically in Ontario during the group therapy visits, for licensure reasons, and to have internet access and a video-enabled device they could access from home or another private location.

Initially eligible participants were invited to a 1-hour pre-group individual interview by a clinician who would be leading their group intervention to confirm their eligibility (including review of inclusion/exclusion criteria, assessment of ACE history, current psychiatric symptoms, and parenting challenges). This objective of this assessment was also to determine a psychiatric formulation and to prepare participants for group participation by reviewing targets for therapy and confidentiality considerations for participation in group therapy, and answering any questions. Groups were created over 12 months on a rolling basis.

Following consent and eligibility assessment, baseline socio-demographic, obstetrical and psychiatric history data were collected via online participant-report questionnaires using an institutionally-approved secure electronic data capture system where participants were sent a personalized link to enter their responses.

Participants were permitted to continue and/or initiate additional forms of psychological and pharmacological treatment while in the study. Participants were provided with tokens of appreciation for completion of final questionnaires ($50 gift card) and interviews ($20 gift card).

### Intervention

The live video-based intervention (MOMBABY) involved twelve weekly two-hour group therapy treatment sessions led by two facilitators (one or both with specialization in perinatal and/or infant mental health) [26]. Each session involved various techniques to target maternal symptoms and the maternal-infant relationship, including psychoeducation, guided mindfulness exercises, guided dyadic play exercises and facilitated space for sharing experiences and mutual support (Appendix A). As is the case in previous in-person MOMBABY groups, there were times when a planned component was not conducted over the course of a given group at facilitator discretion, based on presenting clinical issues. For example, if participants in a certain group cohort did not present with difficulties in a target area (e.g., interpersonal conflict), the corresponding exercise (#8 – Appendix A) could be left out to prioritize interventions focused on the presenting areas of difficulty (e.g., emotion regulation, parenting, mentalization, etc.).

Participants were invited to bring their infants to sessions when able and were encouraged to do so particularly for those sessions involving dyadic exercises. However, when participants were not able to bring their infants, mothers were offered options to engage in an imaginal (i.e., bringing to mind a recent experience of play with their child) or unobserved (at-home practice) version of the play exercise, while still retaining the subsequent in-group reflection with facilitators.

Several adaptations were made from the original in-person intervention previously implemented by members of the study team [26]. Based on early experience with video-based delivery of group therapy at our site [27], the intervention aimed to enroll 4–6 patient participants per group (rather than standard 5–6 in the in-person group). Video-visits were conducted using a healthcare version of Zoom™ that was fully integrated with the hospital electronic medical record (EPIC™ system). This allowed for embedding the use of `share screen’ functions and ‘whiteboard’ features to support discussion and reflection and included distribution of group handouts and virtual group guidelines to participants. This mirrored the activities that had taken place in the previously implemented in-person group, where such materials were handed out to participants on paper, and a physical whiteboard was used in the room. Similarly, facilitators planned to incorporate the dyadic exercises in an inclusive way, working with mothers to decide how best to engage in infant-led play in a virtual setting, but always maintaining the in-group post-play reflection with facilitators.

### Feasibility outcomes

Feasibility of implementation, the primary outcome, was assessed by measuring rates of recruitment, attendance, retention, group component implementation, and technological issues, as well as patient and provider views of the intervention. Data related to attendance and technological issues were tracked weekly by group facilitators, who were also able to add additional written open-ended feedback regarding any benefits, challenges or adaptations required for group component implementation. In addition, a therapist with experience conducting the in-person group (who was not involved in facilitating the sessions) rated n = 36 audiotaped sessions (involving the full 12-sessions of n = 3 randomly selected group cohorts) to determine whether specific components of the group were observed (see Appendix A for intervention fidelity checklist). Patient and provider views were assessed using electronic Likert-scale acceptability questionnaires (Appendices B and C) and participants were offered optional semi-structured post-group acceptability interviews to obtain more detailed feedback (see Appendix D for interview guide). We administered provider acceptability questionnaires after each of the 6 group cohorts. We asked one of the two group facilitators in each group cohort to volunteer to complete the acceptability questionnaire for that group.

### Clinical outcomes

Patient-reported clinical symptoms and parenting-related measures were measured at baseline and at the completion of the 12-week intervention. We used the Edinburgh Postnatal Depression Scale (EPDS), a 10-item scale (range 0–30) validated in the perinatal period to measure depressive symptoms [28], and where a score of ≥ 11 maximizes sensitivity and specificity to detect major depression in postpartum people [29]. We used the Generalized Anxiety Disorder-7 scale (GAD-7), a 7-item scale (score range 0–21) validated perinatally for anxiety symptoms [30]; a score of ≥ 8 represents a useful cutoff for identifying the presence of adult GAD and other anxiety disorders [31, 32]. We also used the PTSD Checklist for DSM5 scale (PCL-5), a 20-item inventory of post-traumatic stress symptoms (score range 0–80) [33]; where cutoffs between 31 and 33 are reasonable for identifying PTSD on the PCL-5 [34].

Additional measures were the Difficulties in Emotion Regulation Scale (DERS), a 36-item scale that measures six facets of emotion regulation (score range 36–180) [35]; the Parenting Stress Index – Short Form (PSI-4-SF), a 36-item scale to identify parent-child problem areas, developed to improve brevity from the 120-item PSI-4 scale [36, 37]; the parent social isolation subscale from the 120-item PSI-4, which had demonstrated pre- to post-group change in evaluation of the in-person group [26]; and the Parental Reflective Functioning Questionnaire (PRFQ), a measure with 3 subscales (each producing a score from 1 to 7), to assess parental reflective functioning or the ability to mentalize one’s infant, a capacity that has been positively associated with parenting quality and attunement [38, 39]. For these additional measures, higher scores suggest greater difficulties, with the exception of the two PRFQ subscales – ‘Certainty About Mental States’ and ‘Interest and Curiosity in Mental States’ where low (and very high) scores suggest greater difficulty [38]. The average time for completion of post-group measures by participants was 7.8 days after group completion.

### Statistical analysis

We described participant baseline characteristics (Table 1), recruitment and retention rates, results from implementation ratings of audio-recordings, and participant and provider responses to acceptability questionnaires using descriptive statistics. We collated comments from providers and participants related to acceptability and experience in the virtual group. Clinical scale scores from pre- to post-group were compared using paired t-tests. Chi square test of proportions was conducted to compare proportion of participants scoring above and below clinical cutoffs for 3 scales (EPDS, GAD7, PCL-5) pre- to post-group.

Analysis of qualitative data from participant semi-structured interview transcripts was informed by the approach outlined in Braun and Clarke’s thematic analysis framework [40]. After initial review of all transcriptions, a deductive approach was employed wherein comments related to our research question (group acceptability) were categorized and grouped into 3 acceptability themes. The aim of this analysis was to provide qualitative data from participant’s group experiences to supplement the quantitative acceptability data obtained from questionnaires. The investigator (EW) who conducted the qualitative interviews and analyzed the data was not involved with facilitation of the groups. The categorization of participant acceptability comments was done by one investigator (EW) and reviewed/confirmed by all other team members. Qualitative data from providers optional written open-ended responses on weekly logs were gathered by one investigator (VW); a second investigator (EW) examined the written responses and categorized those comments related to group acceptability, which was reviewed by all team members.

**Table 1:** Baseline characteristics of n = 31 study participants (presented as n (%), unless indicated otherwise)

	N (%)
**Sociodemographics**	
Mean Age (Standard Deviation) in years	36.5, 3.9 Median = 37
Gender Identity: Woman	> 95%
Non-binary	< 5%
Marital Status: Married, Common-Law, or Cohabitating	27 (87.1%)
Sexual Orientation: Heterosexual	28 (90.3%)
Bisexual and/or Queer	3 (9.7%)
Annual household income ≥$60,000	20 (64.5%)
Racial/Ethnic Identities (not mutually exclusive)	
Black (Caribbean, African, North American)	5 (16.1%)
East Asian (e.g. Chinese, Japanese, Korean)	4 (12.9%)
Indigenous (e.g. First Nations, Inuk/Inuit, Métis)	< 2 (< 6.5%)
Latin American (e.g. Argentinean, Chilean, Salvadorian)	< 2 (< 6.5%)
Middle Eastern (e.g. Egyptian, Iranian, Lebanese)	< 2 (< 6.5%)
South Asian (e.g. Indian, Pakistani, Sri Lankan)	2 (6.5%)
Southeast Asian (e.g. Malaysian, Filipino, Vietnamese)	2 (6.5%)
White (European, North American)	15 (48.4%)
Mixed	< 2 (< 6.5%)
Other	< 2 (< 6.5%)
Completed post-secondary studies (Trades, Diploma, College, University)	28 (90.3%)
Born in Canada	23 (74.2%)
Parity (Median)	1
**Baseline Psychiatric Factors**	
Depression or other mood disorder diagnosis	14 (45.2%)
Anxiety disorder diagnosis	15 (48.4%)
Trauma and stressor-related disorder diagnosis	13 (41.9%)
Current psychiatric medication (any)	15 (48.4%)
Current antidepressant (SSRIs, SNRIs)	13 (41.9%)
Current antipsychotic	< 2 (< 6.5%)
Current anticonvulsant or anxiolytic	< 2 (< 6.5%)

Percentage ranges were used in certain instances to ensure patient anonymity

### Sample size and measures of success

An overall sample size of 25–30 was selected based upon previous literature, suggesting adequacy for participants per group in pilot studies [41]. Our a priori target for success in recruitment was to enroll at least 3–4 groups involving 4–6 participants over 12 months. Targets were also set a priori for participant mean weekly group attendance (≥ 70%), and for retention and completion of study measures (≥ 70% completing group follow-up measures). For success in tracking feasibility of virtual implementation, our a priori criteria were that we would be able to track which components could/could not be implemented virtually and assess whether any components required adaptation for virtual format.

## Results

**Fig. 1:**
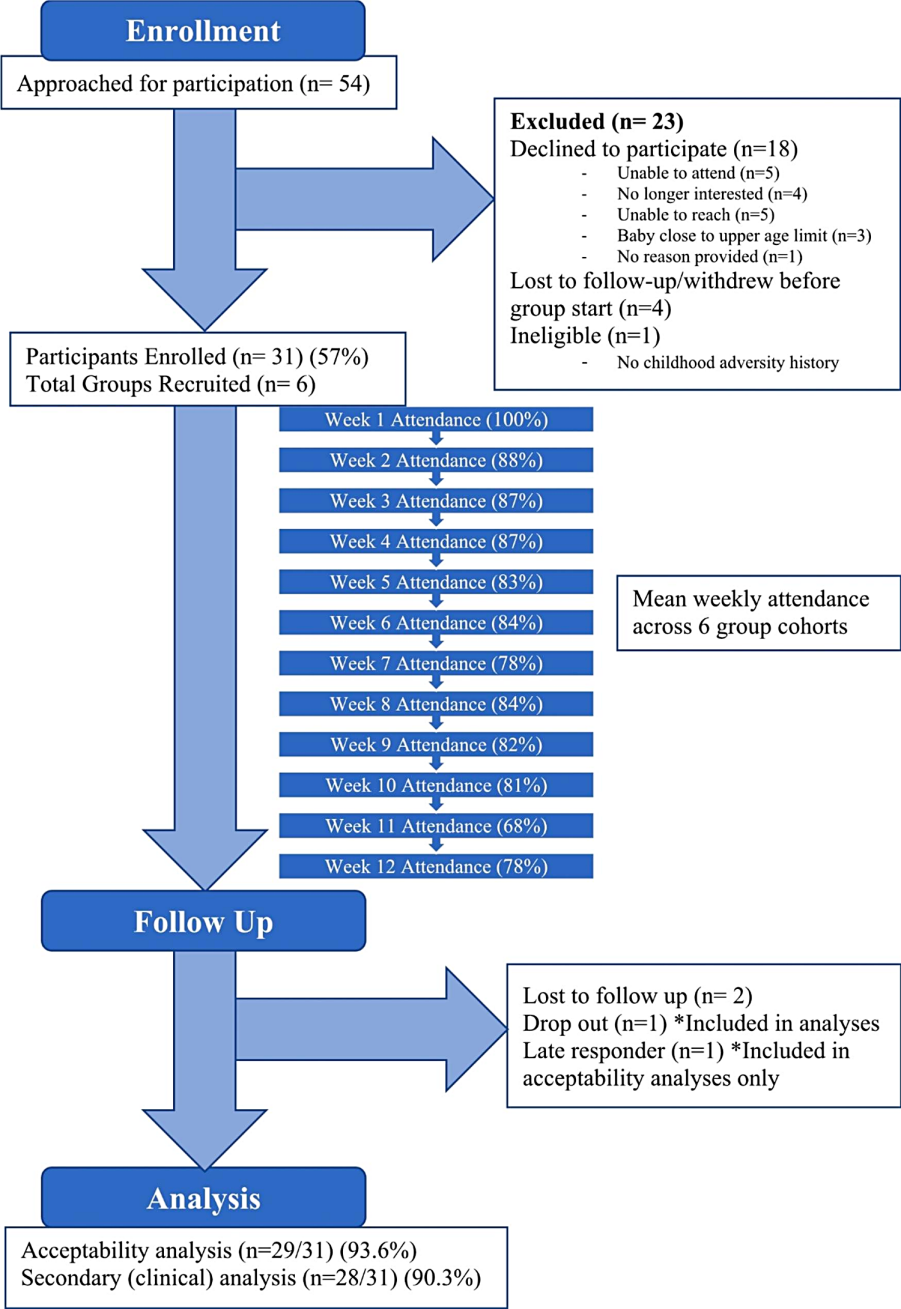
Flowchart of participants’ progress throughout the phases of the evaluation

Of 54 individuals referred to the study, 31 (57%) were enrolled, making up six group cohorts containing 4–6 participants each (Fig. 1). One prospective participant was excluded after clinical interview with their group clinician, as they were inappropriate for the group due to lack of ACE history. The participant was informed by the clinician assessing them that they would not be appropriate for the group, and this clinician also informed their referring provider such that they could be redirected to appropriate services. Twenty-nine (93.6%) participants completed post-treatment questionnaires, but 1 of these participants completed them more than 6 weeks post-group so their responses to the clinical symptom scales were not included in that analysis. One (3.2%) participant withdrew from the study; this occurred after 2 sessions, citing that the group content was not a good fit and that the two-hour sessions were too long.

Participants (> 95% women), mean age 36.5 years (SD 3.9) were mostly primiparous, (Table 1). Most identified as heterosexual (90.3%), about 87.1% were married, common-law, or cohabiting with a partner; 90.3% had completed a post-secondary education program; and 64.5% had an annual household income of more than $60,000. About 74.2% were born in Canada. About 48.4% self-identified as White, 16.1% Black, 12.9% East Asian, and 6.5% for each of South and South-East Asian. About 48.4% reported a past anxiety disorder diagnosis, 45.2% a mood disorder diagnosis, and 41.9% a trauma and stressor-related disorder diagnosis. About 48.4% were taking a psychotropic medication at the time of enrolment.

**Table 2:** Select facilitator and participant responses regarding acceptability of 6 virtual group cohorts

	Facilitators	Participants
Description of technical issues	“Intermittent connectivity, lagging of audio/visual, garbled and choppy audio and freezing was infrequent and brief most of the time.” “[Patients] becoming disconnected, but more often able to reconnect.” “Facilitators and participants joined in 2 different group encounters – able to troubleshoot but delayed start (once).”	“The only difficulty – tech problems. Mine weren’t too bad. But that’s out of peoples’ control and they handled it very well. But for the most part, I did feel like we were in a room - in a sense.”
Perceived benefits to virtual	“Practical: chat option fostered group support, can care for children/infants while on mute, lack of travel, no masks needed making emotional shifts easier to read, sharing digitally and share screen, feasible and safe to reflect, able to stay on if members upset, pets and home surroundings–shared experiences that would otherwise be unknown aspects of [patients].” “Eliminated limited hospital space issue. Less time coordinating room bookings and photocopying.” “Comparable care to in-person.” “Some [patients] still able to join even if sick or conflict with kids arose.” “[Patients] engaged, actively participating, and comfortable. Adjusted well to virtual format.”	“Yes, like unequivocally I would never have been able to do this program if it was in person. I would have quit. Especially in those first 2–3 weeks when I wasn’t quite buying in. I wouldn’t have kept coming. The commute down to WCH - it would have been 4 or 5 hours of my day. Got my kids to childcare, got myself there, I could not have done a 4–5 hour per day commitment for 12 weeks. But 2 hours online was doable. I think that the exception should be in person. I think the online piece is what made it so accessible and removed a lot of barriers.” “Well one thing is that it allows you to kind of experience this in your home setting where maybe you are more likely to face situations like your baby crying. Learning how to practice while holding my baby at home, made me think that the next time, without the group, let’s try this in the home setting.” “The virtual access was huge for me. Because I definitely would not have been able to access with everything happening. I would not have been able to be part of the group if it was happening in person. The virtual was huge access for me as a new mom, first time mom, c section mom, and struggling with all of the postpartum challenges I had.”
Perceived drawbacks to virtual	“Slower to warm -- less spontaneous engagement from [patients] initially and still throughout.” “Unable to enhance emotional connection and community through nonverbal communication. Body language more difficult to read.” “Mothers multitasking (benefit and drawback?)” “Less satisfying to say goodbye over Zoom” “[Patients] distracted by other things such as device notifications.” “One [patient] commented stressful to be on camera, distracted looking at others, hard to stay present.” “[Dyadic exercise] very adapted, not able to observe interaction but still useful.”	“One drawback I would actually say is I feel like in-person we would have more time to increase those connections with the other members, because online it’s like ‘okay we’re done, goodbye’. I feel like it would have been nice to continue a chat – not so much group focused, in terms of therapy – just that social aspect to it.” “I do kind of wish there had been one or two in-person meetings. I love virtual space and I understand why it wasn’t possible. In some ways, the virtual space allowed the group to be more at ease with each other, seeing each other in our homes. But I did miss the opportunity to meet some of the participants in person.” “I thought that in one way, it was very convenient for it to be online. But on the other hand, I think - in an ideal world where I didn’t have to work, and we weren’t in covid, I could just like go into these sessions and get a babysitter or something - being in person would have been awesome. But being that it was covid and I had to work, there was no other way that I could have done it. Even if I was on mat leave without covid it wouldn’t have been easy to go.”

The mean weekly attendance rate for the groups was 83.3% (range 68–100%). In independent ratings, all 12 group components were observed at least once, with all except 2 components observed in all 3 rated group cohorts (Appendix A). The component ‘Discussion and reflection on ‘attachment styles’ [component 6a + b] was only observed in 1 of 3 rated groups. The dyadic play exercise [component 7] was only observed in 2 of the 3 rated groups, with one group facilitator electing to leave it out based on the presenting clinical issues of this groups’ members. In the two group cohorts where the dyadic play exercise was introduced, participants were involved in ad-hoc collaborative decision making with facilitators to determine how best to integrate the play exercise by video. One cohort elected to engage in the dyadic play outside of the group session for various practical reasons, including those related to childcare arrangements, with a plan for subsequent reflection and discussion during the following session. The other cohort elected to try the exercise during group session; participants decided that cameras be turned off for the play portion of the session so that screens would not be a distraction for children.

No adverse events or serious adverse events were reported. All respondents to the acceptability questionnaire (n = 29/29) agreed or strongly agreed that the virtual group was easy to access, beneficial, improved their ability to access care, and reduced barriers to participation such as transportation and child-care (Appendix B). All but one participant (96.6%) felt the virtual group format should remain as an option, with the remaining participant neither agreeing nor disagreeing with this statement. About 82.8% (n = 24/29) of participants agreed or strongly agreed that they were as comfortable engaging with virtual group psychotherapy as an in-person group; one participant disagreed with this statement, and 4 (13.8%) neither agreed nor disagreed. Five (17.3%) participants encountered technical problems that interfered with group access at some point during group participation. We approached 21 participants in order to obtain 20 participants for the post-group acceptability interview, with 1 participant declining. Deductive thematic analysis of participant interview transcripts related to our research focus of participant acceptability identified three themes. Select participant comments related to these 3 themes - perceived benefits, drawbacks, and technical issues arising with virtual group – are shown in Table 2.

Acceptability questionnaires were completed by one facilitator from each of the 6 study groups, for 6 responses from 3 unique facilitators (facilitators ran multiple groups). All indicated that delivering the groups virtually was easy, a positive experience, enabled a similar quality of care to that provided in-person, and allowed them to sufficiently address patients’ clinical needs (Appendix C). All indicated that they would be happy to facilitate virtually in future. Facilitators identified technical problems that interfered with facilitation during 4/6 (66.7%) group cohorts (Appendix C). Optional written open-ended post-group feedback from facilitators was reviewed for comments related to acceptability. Select facilitator comments illustrating perceptions of acceptability are shown are in Table 2, presented alongside comments from participants.

**Fig. 2:**
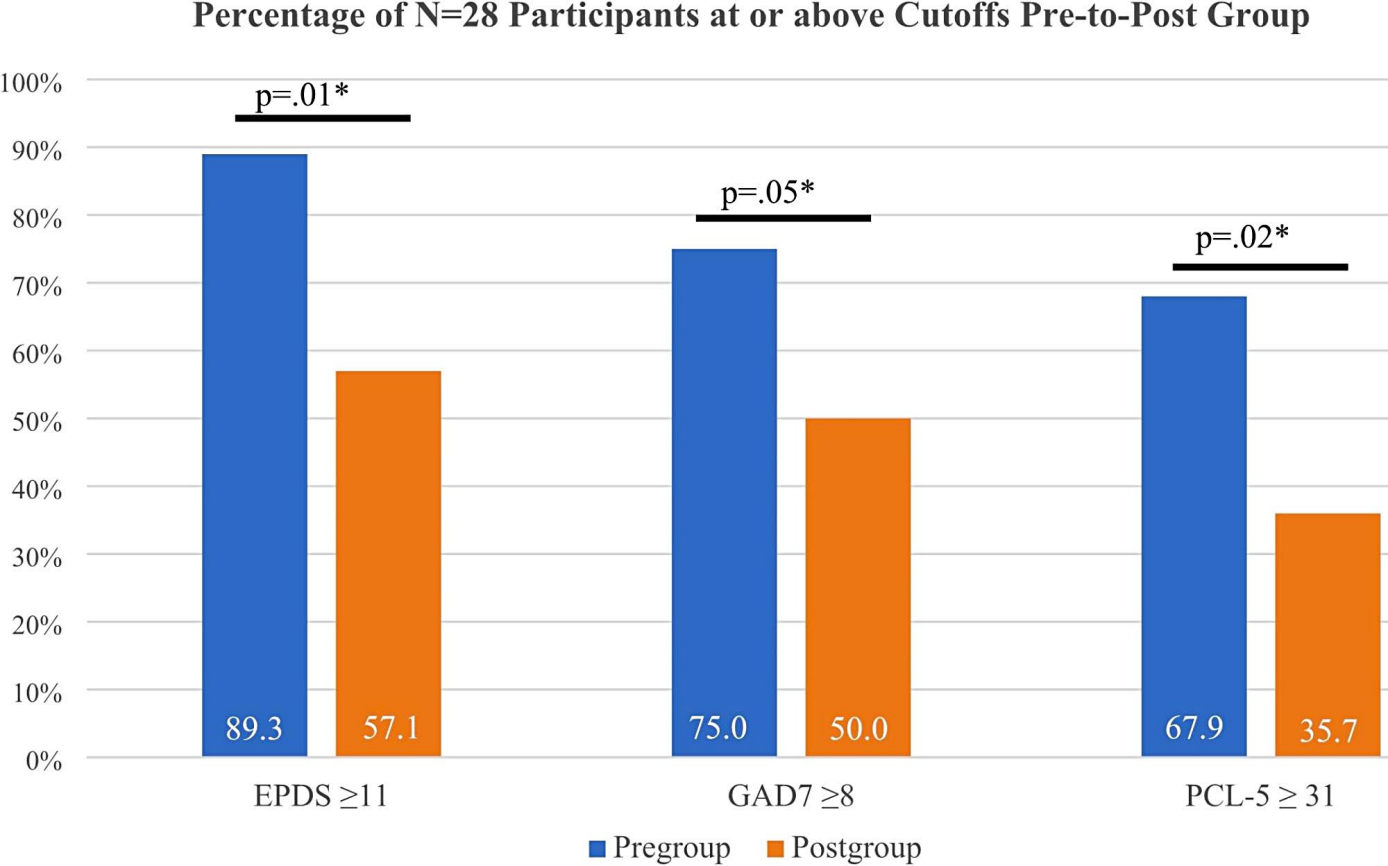
Percentage of N = 28 participants at or above cut-offs before and following virtual group. *= significant at p ≤ 0.05 level. EPDS, Edinburgh Postnatal Depression Scale; GAD-7, Generalized Anxiety Disorder-7 scale; PCL-5, PTSD Checklist for DSM5 scale

Participant EPDS and PCL-5 scores decreased significantly from pre- to post-group, from 14.6 (Standard Deviation, SD, 4.2) to 11.8 (4.2)(*Mean difference, MD -2.86, 95% CI -4.75 to -0.96*) and 35.3 (18.9) to 27.1(16.7)(*Mean difference, MD -8.14, 95% CI -13.88 to -2.40*), respectively; GAD-7 scores decreased as well, but not significantly so (*Mean difference, MD -1.36, 95% CI -3.33 to 0.61*) (Table 3). The percentage of participants with clinically significant symptoms of depression (EPDS ≥ 11) decreased from 89.3 to 57.1% post-group (*X*^2^ (1, N = 28) = 7.4, p = 0.01), the percentage with clinically significant symptoms of anxiety (GAD-7 ≥ 8) decreased from 75.0 to 50% post-group (*X*^2^ (1, N = 28) = 3.7, p = 0.05) and the percentage with clinically significant symptoms of PTSD (PCL-5 ≥ 31) decreased from 67.9 to 35.7% post-group (*X*^2^ (1, N = 28) = 5.8, p = 0.02) (Fig. 2). Scores for mothers’ emotion regulation, parenting stress, parental reflective functioning, and parent social isolation also improved, but not significantly so (Table 3).

**Table 3:** Clinical and parenting-related scale scores at baseline and post-group follow up (n = 28), mean (SD) and mean differences with 95% confidence interval (CI) presented

Clinical Symptoms	Pregroup Mean (SD)	Postgroup Mean (SD)	Mean Difference (95% CI)
EPDS	14.6 (4.2)	11.8 (4.2)	-2.86 (-4.75 to -0.96)
GAD-7	10.0 (4.5)	8.6 (4.7)	-1.36 (-3.33 to 0.61)
PCL-5	35.3 (18.9)	27.1 (16.7)	-8.14 (-13.88 to -2.40)
DERS	98.4 (23.3)	93.9 (19.8)	-4.54 (-13.78 to 4.70)
Parenting-Related Functioning			
PSI-SF Total	87.9 (20.5)	86.3 (21.5)	-1.57 (-7.87 to 4.73)
PSI – Social Isolation Subscale	18.7 (4.8)	18.1 (5.3)	-0.61 (-2.03 to 0.82)
PRFQ Subscales			
Pre-Mentalizing Modes	1.8 (0.7)	1.9 (0.8)	0.06 (-0.22 to 0.34)
Certainty About Mental States	3.5 (1.0)	3.6 (1.0)	0.16 (-0.19 to 0.52)
Interest and Curiosity in Mental States	5.8 (0.9)	6.1 (0.7)	0.29 (-0.04 to 0.61)

EPDS, Edinburgh Postnatal Depression Scale; GAD-7, Generalized Anxiety Disorder-7 scale; PCL-5, PTSD Checklist for DSM5 scale; DERS, Difficulties in Emotion Regulation Scale; PSI-SF, Parenting Stress Index – Short Form; PSI, Parenting Stress Index (Social Isolation Subscale only); PRFQ, Parental Reflective Functioning Questionnaire

## Discussion

To address common barriers to accessing in-person mental health care faced by postpartum individuals, we piloted a virtual adaptation of a maternal-infant therapy group tailored for mothers with psychiatric illness and a history of childhood adversity. In a sample diverse in terms of sexual orientation and racial and ethnic identity – with about one quarter of participants born outside of Canada, we found that the live video-based intervention was feasible to implement with good fidelity to the planned model. The video-based group was acceptable to participants and providers, and we demonstrated a high adherence to study measures that suggests proceeding to larger scale evaluation would be feasible. While this was a pilot, non-comparative study, the maternal symptom improvement from pre to post group in depressive and post-traumatic symptom domains was also a promising finding.

Aligned with other studies reporting better engagement and completion rates for virtual vs. in-person postpartum psychotherapy interventions [42], group attendance (mean 83%) was high, and better than in the evaluation [26] of the in-person group (68%). Numerous participants commented on how the virtual modality reduced barriers as new mothers (child-care, commute, etc.) as has been cited in other evaluations of virtual postpartum mental health care [43]. Reported rates of technology “glitches” were at levels similar to that observed in other comparable interventions [44], and acceptability was high. While this was not a comparative study powered for efficacy results, the direction of the clinical outcomes comparing post-group to pre-group is consistent with evidence on parent-child interventions for older (non-infant) children modified for online delivery that showed improved outcomes [45, 46], as well as with evidence on mindfulness-based programs, emotion-regulation training, peer support groups, and other parenting interventions successfully adapted for online administration [47–51].

The live video-based delivery model did require some key adaptations, most notably related to the dyadic exercise. While this exercise was implemented as in in-person groups, when dictated by presenting clinical issues, it required modification. In this case, in the virtual adaptation of the exercise, facilitators did not have the opportunity to directly observe the play exercise within the dyad in any group cohort. What was retained was the use of this experiential play exercise as a tool to facilitate self-reflection, mentalization and insight-oriented discussion. Participants reported high acceptability of this approach. However, it will be important in future to understand the potential impacts of this adaptation on parent-infant and infant outcomes through (a) comparison to an in-person condition that allows for the direct observation more fully, and (b) adding more objective assessment measures of the parent-child relationship and infant outcomes. It may also be worth additionally exploring alternative workarounds that could be used for adapting the dyadic exercise to the virtual format and still allow facilitator observation. For example, digital features such as ‘hide self’ view for participants and ‘pinning’ the clinician into view could be used, such that children are not distracted by their own play or that of the other families. Two group components (6 + 7) were not observed in all group cohorts, with group co-facilitators electing not to include this material in certain groups based upon group need and composition. This is consistent with the process of inclusion in the in-person group and was not related to shift to virtual setting.

Other important feedback about the virtual adaptation was that several participants expressed a desire for more connection with co-participants, with some suggesting an opportunity to connect in-person or outside of group sessions would be a valuable addition. Aligned with this feedback, a measure of parent social isolation that had improved to a significant degree in a previous study of the in-person group [26], did not improve as much in the virtual groups. This may reflect a drawback to virtual group participation, wherein participants do not experience equivalent improvement in feelings of social isolation when compared with in-person groups. Together, these findings suggest that providers might explore hybrid models of care (post-pandemic) or provide opportunities for in-person meetings to improve group connectedness while maintaining the practical benefits of primarily virtually delivered care.

Finally, there are some limitations to the study to be considered as we move toward larger scale implementation and evaluation. We only included participants with access to internet from their homes or another private location on a personal device. No participants approached for participation expressed a lack of access to internet and a video-enabled device, but these concerns are relevant to ensuring inclusion of marginalized populations. Providing devices/internet access in future should be considered, as has been done in other evaluations of online parenting interventions for new mothers [49]. We also only used self-report measures rather than rater-reported to measure preliminary clinical effects of group participation, and we did not track treatments that participants may have engaged with during the group (e.g. medications started after baseline, individual psychotherapy). A future controlled study – with comparison to an in-person group - could incorporate structured clinical interviews and observed parent-child interaction for more comprehensive outcome assessment. It would be important in future comparative research for concomitant treatments (including psychotherapy and medication) to be carefully tracked. A future larger evaluation could also consider a quantitative measure of childhood adverse experience history (e.g. the 10 item Adverse Childhood Experiences Questionnaire [52]) to understand whether the group is similarly effective across different levels of ACE. Given participant feedback around social connection, it may be useful to include an assessment of group factors (e.g. group cohesion) in a future evaluation - such factors can be negatively impacted by a shift to virtual environment and may relate to perceptions of social isolation [53]. Further, collection of demographic data about group facilitators would be valuable to collect in future studies. Finally, though this pilot feasibility study was not intended to be powered to the detection of pre/post effects on parenting, we found that measures of parenting stress and parental reflective functioning trended towards improvement, which is promising. A future appropriately powered comparative study would better allow us to comment on the intervention’s effect or lack of effect on parenting status, including with a longer outcome window, as changes in parenting behaviour may be captured at 6 months follow up, even when not significant immediately post-intervention [54].”

## Conclusions

In summary, this study supports the feasibility of implementing an adapted live-video based mother-infant psychotherapy group for mothers with ACE, with the most notable modification being related to how to implement mother-infant dyadic exercises in the context of virtual care provision. A future comparative trial is warranted to definitively evaluate efficacy as well as effectiveness and implementation considerations for different patient subgroups.

### Electronic supplementary material

Below is the link to the electronic supplementary material.

**Supplementary Material 1:** Appendices A-D

**Supplementary Material 2:** Baseline Characteristics of Interviewed vs. Not Interviewed Participants

## Data Availability

Data used and analysed for the current study are available from the corresponding author upon reasonable request.

## Abbreviations

ACE: Adverse Childhood Experiences
EPDS: Edinburgh Postnatal Depression Scale
GAD-7: Generalized Anxiety Disorder-7 scale
PCL-5: PTSD Checklist for DSM5 scale
DERS: Difficulties in Emotion Regulation Scale
PSI-4: Parenting Stress Index
PSI-4-SF: Parenting Stress Index – Short Form
PRFQ: Parental Reflective Functioning Questionnaire
