# Supplementary material for: A virtual mother-infant postpartum psychotherapy group for mothers with a history of adverse childhood experiences: open-label feasibility study
Source: BMC Psychiatry. 2023 Dec 18;23:950. doi: 10.1186/s12888-023-05444-x (PMC10726650; doi:10.1186/s12888-023-05444-x)
Supplement: Supplementary file 1 — Supplementary Material 1: Appendices A-D [file 12888_2023_5444_MOESM1_ESM.docx]

**Appendix A – Intervention Fidelity Checklist with Group Components**

Rater scoring of virtual implementation of group components in audio recordings of the 3 rated group cohorts (X=implemented)

| Group Component | Implemented in Group 1 | Implemented in Group 2 | Implemented in Group 3 |
| --- | --- | --- | --- |
| 1. Introduction to, and description of, mindfulness practice | X | X | X |
| 2. Guided mindfulness exercise with group | X | X | X |
| 3. Psychoeducation regarding emotional dysregulation | X | X | X |
| 4. Reviewed strategies for emotion regulation | X | X | X |
| 5a. Introduced/discussion of parenting styles | X | X | X |
| 5b. Reflection of group members regarding above | X | X | X |
| 6a. Introduced/discussion of attachment styles | X |  |  |
| 6b. Reflection of group members regarding above | X |  |  |
| 7. Facilitated a virtual ‘dyadic play exercise and subsequent reflection/discussion |  | X | X |
| 8. Discussion of interpersonal dynamics in conflict | X | X | X |
| 9. Discussion re: impacts of early trauma/adversity as related to parenting | X | X | X |
| 10. Facilitated space for members to share experience and provide mutual support | X | X | X |

**Appendix B**

Acceptability questionnaire completed after group by n=29 participants (unless otherwise specified), presented as n(%) for the category

| Questionnaire Item |  | Strongly Disagree | Disagree | Neither Agree nor Disagree | Agree | Strongly Agree |
| --- | --- | --- | --- | --- | --- | --- |
| 1. I encountered technical problems that interfered with accessing this virtual group. | | 13 (44.8) | 8 (27.6) | 3 (10.4) | 4 (13.8) | 1 (3.5) |
| 2. Overall, I found this virtual group easy to access.^1^ | | 0 | 0 | 0 | 9 (32.1) | 19 (67.9) |
| 3. Participating in this virtual psychotherapy group was a positive and supportive experience. | | 0 | 0 | 1 (3.5) | 7 (24.1) | 21 (72.4) |
| 4. I felt comfortable sharing information about myself in a virtual psychotherapy group. | | 0 | 1 (3.5) | 0 | 9 (31.0) | 19 (65.5) |
| 5. I felt as comfortable engaging with the virtual psychotherapy group as an in-person group. | | 0 | 1 (3.5) | 4 (13.8) | 7 (24.1) | 17 (58.6) |
| 6. It was helpful to access the virtual psychotherapy group from home/anywhere with a secure connection. | | 0 | 0 | 0 | 7 (24.1) | 22 (75.9) |
| 7. I benefited from the information and therapy interventions in the virtual psychotherapy group. | | 0 | 0 | 0 | 8 (27.6) | 21 (72.4) |
| 8. I felt supported and heard by the therapists. | | 0 | 1 (3.5) | 0 | 5 (17.2) | 23 (79.3) |
| 9. I was satisfied with the level of engagement of the other virtual group members. | | 0 | 2 (6.9) | 0 | 6 (20.7) | 21 (72.4) |
| 10. I benefited from the input of other virtual group members. ^1^ | | 0 | 1 (3.6) | 0 | 7 (25) | 20 (71.4) |
| 11. I was satisfied with my own level of engagement in the virtual psychotherapy group. ^1^ | | 1 | 3 (10.7) | 1 (3.6) | 5 (17.9) | 18 (64.3) |
| 12. I feel that I was able to learn the necessary material and skills through the virtual group. | | 0 | 1 (3.5) | 3 (10.3) | 7 (24.1) | 18 (62.1) |
| 13. Having the virtual group platform improved my ability to access care. | | 0 | 0 | 0 | 9 (31.0) | 20 (69.0) |
| 14. The virtual group format should remain as an option for participants. | | 0 | 0 | 1 (3.5) | 6 (20.7) | 22 (75.9) |
| 15. The virtual group format was helpful to reduce transportation, child-care, other barriers to participation.^1^ | | 0 | 0 | 0 | 4 (14.3) | 24 (85.7) |

1 not all participants answered this question (n=28 responders for 4 marked questions)

**Appendix C**

Acceptability questionnaire completed by three unique co-facilitators following n=6 unique group cohorts, presented as n(%)

| Questionnaire Item |  | Strongly Disagree | Disagree | Neither Agree nor Disagree | Agree | Strongly Agree |
| --- | --- | --- | --- | --- | --- | --- |
| 1. I encountered technical problems that interfered with facilitating this virtual group. | | 1 (16.7) | 1 (16.7) | 0 | 4 (66.7) | 0 |
| 2. Overall, I found this virtual group easy to deliver. | | 0 | 0 | 0 | 3 (50.0) | 3 (50.0) |
| 3. Facilitating this virtual psychotherapy group was a positive experience. | | 0 | 0 | 0 | 0 | 6 (100) |
| 4. I felt comfortable using the virtual visit technology to provide group-based clinical care. | | 0 | 0 | 0 | 1 (16.7) | 5 (88.3) |
| 5. The participants were adequately prepared to participate in their group video visits. | | 0 | 0 | 0 | 2 (33.3) | 4 (66.7) |
| 6. The quality of care I could provide through group video visits was similar to those that are conducted in person. | | 0 | 0 | 0 | 6 (100) | 0 |
| 7. Group video visits enable me to sufficiently address patients’ clinical needs. | | 0 | 0 | 0 | 2 (33.3) | 4 (66.7) |
| 8. I felt satisfied with the care I provided to the group virtual visit. | | 0 | 0 | 0 | 0 | 6 (100) |
| 9. I was satisfied with the level of engagement of the virtual group members. | | 0 | 0 | 0 | 2 (33.3) | 4 (66.7) |
| 10. I spent the same amount of time on the virtual group visit (including preparation and planning) as I would have for an in-person group. | | 0 | 2 (33.3) | 0 | 3 (50.0) | 1 (16.7) |
| 11. I spent the same amount of effort (including preparation and planning) as I would have for an in-person group. | | 0 | 1 (16.7) | 0 | 4 (66.7) | 1 (16.7) |
| 12. I would be happy to facilitate a virtual group again. | | 0 | 0 | 0 | 0 | 6 (100) |
| 13. In the future, I would like to have the option of being able to facilitate a virtual group. | | 0 | 0 | 0 | 0 | 6 (100) |
| 14. In the future, if I had the choice between an in-person group and a virtual group, I would choose to facilitate the virtual group. | | 0 | 0 | 3 (50.0) | 2 (33.3) | 1 (16.7) |

A**ppendix D** - Qualitative semi-structured interview guide

1. What influenced your decision to participate in the group?
2. What were your expectations of the group?
   1. In which ways did the group match/not match your expectations?
   2. What was satisfying/not so satisfying about the group intervention?
3. What is your previous group experience (professional and casual):
   1. How different was this group?
   2. Would you like to have had more interaction and feedback from the other group members? Why?
4. How did it feel to have your individual therapist/psychiatrist facilitating or observing the group (if applicable)?
5. What was most helpful about the group?
6. What was least helpful about the group?
7. What would you change?
8. What did you gain?
9. What was the group process like for you?
10. What were the major changes you experienced from the group?
11. What have we missed and what else would like to say?
12. Should the virtual group format continue to be offered to future participants (i.e. beyond the pandemic)?
