# Supplementary material for: A virtual mother-infant postpartum psychotherapy group for mothers with a history of adverse childhood experiences: open-label feasibility study
Source: BMC Psychiatry. 2023 Dec 18;23:950. doi: 10.1186/s12888-023-05444-x (PMC10726650; doi:10.1186/s12888-023-05444-x)
Supplement: Supplementary file 2 — Supplementary Material 2: Baseline Characteristics of Interviewed vs. Not Interviewed Participants [file 12888_2023_5444_MOESM2_ESM.docx]

**Supplementary Table**

Baseline characteristics of n=20 interviewed and n=11 non-interviewed study participants (presented as n (%), unless indicated otherwise)

|  | Interviewed  N(%) | Not Interviewed  N (%) |
| --- | --- | --- |
| Sociodemographics |  |  |
| Mean Age (Standard Deviation) in years | 36 (5) | 37 (2) |
| Gender Identity: Woman | > 18 (> 90%) | 11 (100%) |
| Marital Status: Married, Common-Law, or Cohabitating | 16 (80%) | 11 (100%) |
| Sexual Orientation: Heterosexual | 18 (90%) | > 9 (> 90%) |
| Completed post-secondary studies | 17 (85%) | 11 (100%) |
| Born in Canada | 14 (70%) | 9 (82%) |
| Annual household income ≥$60,000 | 13 (65%) | 7 (64%) |
| Racial/Ethnic Identities (not mutually exclusive) |  |  |
| Black (Caribbean, African, North American) | 4 (20%) | < 2 (< 18%) |
| East Asian (e.g Chinese, Japanese, Korean) | 3 (15%) | < 2 (< 18%) |
| Indigenous (e.g First Nations, Inuk/Inuit, Métis) | < 2 (< 10%) | 0 |
| Latin American (e.g. Argentinean, Chilean, Salvadorian) | < 2 (< 10%) | 0 |
| Middle Eastern (e.g. Egyptian, Iranian, Lebanese) | 0 | < 2 (< 18%) |
| South Asian (e.g. Indian, Pakistani, Sri Lankan) | 2 (10%) | 0 |
| Southeast Asian (e.g. Malaysian, Filipino, Vietnamese) | 2 (10%) | 0 |
| White (European, North American) | 8 (40%) | 7 (63%) |
| Mixed | < 2 (< 10%) | 0 |
| Other | 0 | < 2 (< 18%) |
| Baseline Psychiatric Factors |  |  |
| Depression or other mood disorder diagnosis | 10 (50%) | 4 (36%) |
| Anxiety disorder diagnosis | 8 (40%) | 7 (63%) |
| Trauma and stressor-related disorder diagnosis | 9 (45%) | 4 (36%) |
| Current psychiatric medication (any) | 9 (45%) | 6 (55%) |
| Current antidepressant (SSRIs, SNRIs) | 8 (40%) | 5 (45%) |
| Current antipsychotic | < 2 (< 10%) | 0 |
| Current anticonvulsants or anxiolytic | < 2 (< 10%) | 0 |
| Mean (SD) Edinburgh Postnatal Depression Scale score | 15.1 (3.4) | 13.4 (5.7) |
| Mean (SD) Generalized Anxiety Disorder-7 scale score | 10.0 (4.3) | 10.3 (5.9) |
| Mean (SD) PTSD Checklist for DSM5 scale score | 37.6 (17.4) | 29.2 (22.7) |
|  |  |  |
| Group Attendance Rate –12-week average (%) | 85% | 78% |

Percentage ranges were used in certain instances to ensure patient anonymity.
